# Aerodigestive Tract Burn from Ingestion of Microwaved Food

**DOI:** 10.1155/2013/781809

**Published:** 2013-03-31

**Authors:** Michael Silberman, Rebecca Jeanmonod

**Affiliations:** St. Luke's University Hospital and Health Network, 801 Ostrum Street, Bethlehem, PA 18015, USA

## Abstract

Aerodigestive tract burns represent a rare but potentially devastating injury pattern throughout the world. Although the majority of these injuries do not require intervention, these burns have the potential for poor outcomes. Traditionally this disease has been caused by superheated gases found in explosions or fire-related injury. However, as technology advances, it brings novel methods for injury that require physician awareness of potential hazards. We describe a case of laryngeal and esophageal thermal burn caused by a microwave heated food bolus.

## 1. Case Presentation

A 79-year-old male presented to the emergency department (ED) with symptoms of dysphagia. The patient related that 5 hours prior to admission, he had eaten a piece of lasagna that he had microwaved. The patient reported that the food had gotten extremely hot, and rather than spitting it out, he swallowed the food bolus immediately. After swallowing, he felt a burning sensation down his “entire throat.” The patient had had increasing difficulty with swallowing since this event, and noted that he could no longer swallow water. This prompted him to come to the emergency department. The patient also complained of a persistent cough, although he denied any dyspnea. He had no other complaints and no significant past medical history.

On physical exam the patient had normal vital signs but appeared clearly uncomfortable. The patient's ear, nose, and throat exam showed pharyngeal erythema as well as obvious difficulty with oral secretions including some drooling and a hoarse, “gargle” sounding voice. The patient's neck was supple with no palpable masses. The patient's lungs were clear to auscultation throughout with no evidence of stridor, wheeze, rhonchi, rales, or crepitance. The remainder of his physical exam was unremarkable. 

The patient's ability to handle his secretions worsened during his ED stay, until he was unable to swallow any saliva. Gastroenterology was consulted and the patient had an emergent esophagogastroduodenoscopy (EGD) performed. The EGD showed that there was significant inflammation of the larynx and vocal cords most consistent with a thermal burn. Additionally, there was erythema throughout the length of the esophagus with mild swelling, and some nonobstructing food debris was found in the distal esophagus. The patient received 10 mg dexamethasone for airway edema while in the gastroenterology procedure room following EGD. He was extubated after the procedure and was transferred to the intensive care unit for continued airway monitoring. The patient did well and was discharged 48 hours later with no residual breathing difficulty, dysphagia, or odynophagia, with a diagnosis of laryngeal and esophageal thermal burn.

## 2. Discussion

Burns are a leading cause of accidental injury and death in the United States, and each year approximately 1 million people in the US seek medical care for burns [1–4]. Although the vast majority of injuries do not require hospitalization, severe burns can lead to significant morbidity and death [1–4]. Despite increasing understanding of burn care and ventilator assessment, injury resulting in laryngeal and tracheal damage remains a leading cause of death in adult burn victims [1, 2, 5]. These upper airway burns have historically been caused by inhalation of either superheated gasses or thermally heated debris. Significant burns with resultant airway edema due to voluntary ingestion of edible materials were not reported at all in the literature until microwave ovens became commonplace in homes in developed nations.

Since their introduction in 1947, microwaves have been recognized for their convenience, but several reports have raised concern that there is a higher risk of oropharyngeal burns with foods heated by microwave ovens. In order to understand this risk, one needs to understand how microwaves work. A microwave oven cooks food through dielectric heating. In this process, microwave-type electromagnetic radiation rotates and heats polarized molecules in food [6]. Because fats and sugars are less polar than water, microwave heating is less efficient on these food types [7]. Additionally, these substances have a lower heat capacity and a higher vaporization temperature than water, allowing them to attain temperatures far above the boiling point of water when exposed to microwave radiation [7]. Since microwaves do not rely on ambient increase in temperature to cook foods and rather on the properties of the food items themselves, heterogeneous foods display differential heating, often developing “hot spots.” Food-related thermal burns may result from the ingestion of these foods because consumers overlook the differential temperature gradients within foods and between the food and its container [6].

The approach to upper airway burns secondary to ingestion of a hot food bolus is similar to that of burns secondary to inhalation injury. Airway maintenance is critical, and supplemental oxygen should be provided as needed [8]. Upper airway edema following a burn-related injury can occur rapidly, and many develop complete airway obstruction with no clinical means to determine which patient will do so [8]. Intubation should not be delayed if respiratory distress is present or anticipated in a burn victim. Common signs of impending respiratory distress shared by both inhalation and ingestion burn injury include persistent cough, stridor, wheezing, hoarseness, blistering or edema of the oropharynx, hypoxia, or hypercapnia [8].

Although airway support is the first priority in all upper airway burns, burns secondary to a food bolus have the added risk of esophageal injury. There are very few cases of these burns reported in the literature [9, 10]. Although there is no specific evidence in the literature regarding management of food-bolus related esophageal burns, there is much information available on chemical esophageal burns from caustic ingestions and thermal esophageal burns related to atrial ablation therapy, which may be extrapolated to these cases. Thermal burns related to atrial ablation therapy have no validated grading system, but the likelihood of adverse outcomes appears directly correlated with the depth of thermal injury [4]. For caustic burns, as well, there is literature to suggest that depth of burn and presence of perforation are the greatest indicators of clinical outcome [11]. A grading system for caustic ingestion exists that rates mucosal damage from grade 0 to 3, 0 being normal tissue, 1 showing mild edema, 2A showing superficial mucosal ulceration, 2B showing deep focal or circumferential ulceration, 3A showing focal mucosal necrosis, and 3B showing extensive mucosal necrosis [11]. This grading system has been shown to be of prognostic value. Patients with grade 1 and 2A burns have an excellent prognosis without significant acute morbidity or subsequent stricture formation [11]. Patients with grades 2B and 3A develop strictures in 70 to 100 percent of cases [11]. Grade 3B carries a 65-percent early mortality risk and the need for esophageal resection with colonic or jejunal interposition in most cases due to depth of burn [11]. Patients with caustic burns are at increased risk for both cancer and stricture formation mandating followup with EGD screening several years following injury, although this increased risk has not been defined with thermal injury [11]. 

Although treatment based on this scoring system has not been prospectively validated, protocols based on observational data have been utilized to guide patient care [11]. Patients with grade 1 or 2A injury require no specific therapy beyond supportive care [11]. A liquid diet may be initiated and the patient can be advanced to a regular diet in 24 to 48 hours [11]. Patients with grade 2B or 3 injuries should have nasoenteric tube feeding initiated after 24 hours to rest the esophagus [11]. Oral liquids are allowed after the first 48 hours if the patient is able to swallow saliva [11]. Because the majority of deaths are due to esophageal perforation and resultant mediastinitis, EGD within 24 hours is mandated in all caustic injuries [11]. Patients with esophageal burns from ablation therapy are also recommended to have EGD in one week to assess for burn healing [4]. 

Steroid use is contraindicated for esophageal chemical burns, as it has been shown to increase mortality [10]. The utilization of steroids in thermal burns to the larynx and esophagus has not been firmly established [1, 3–5, 8]. Proton pump inhibitor therapy has been shown to improve esophageal healing for patients with caustic esophageal injuries and is also recommended in patients with thermal esophageal injury [4, 12]. Because of risk for future stricture and malignancy with caustic injuries, these patients follow with gastroenterology indefinitely. Long-term outcomes have not been determined for thermal burn patients due to a paucity of reported cases. 

## 3. Conclusion

Ingestion of hot food boluses can cause both airway and esophageal thermal burns. Airway issues need to be treated aggressively, with early intubation if the patient exhibits any respiratory distress. Patients should also undergo EGD to evaluate the extent of esophageal injury to help guide both treatment and followup. Proton pump inhibitor therapy may aid in the healing process of these injuries [4, 12]. Steroid use is controversial.
